# Medium-Range Narratives as a Complementary Tool to Principle-Based Prioritization in Sweden: Test Case “ADHD”

**DOI:** 10.1007/s11673-018-9884-3

**Published:** 2018-12-05

**Authors:** Pier Jaarsma, Petra Gelhaus

**Affiliations:** 1 Division of Health Care Analysis, Department of Medical and Health Sciences (IMH), University of Linköping, Malmstigen 13, 58941 Linköping, Sweden; 2 Institute for Ethics, History and Philosophy of Medicine, University of Muenster, Muenster, Germany; 3 Borghamn, Sweden

**Keywords:** ADHD, Ethical framework, Medium-range narratives, Prioritization, Swedish healthcare system

## Abstract

In this paper, for the benefit of reflection processes in clinical and in local, regional, and national priority-setting, we aim to develop an ethical theoretical framework that includes both ethical principles and medium-range narratives. We present our suggestion in the particular case of having to choose between treatment interventions for attention deficit hyperactivity disorder (ADHD) and treatment interventions for other conditions or diseases, under circumstances of scarcity. In order to arrive at our model, we compare two distinct ethical approaches: a generalist (principles) approach and a particularist (narratives) approach. Our focus is on Sweden, because in Sweden prioritization in healthcare is uniquely governmentally regulated by the “ethics platform.” We will present a (fictional) scenario to analyse the strengths and weaknesses of the generalist principled perspective of the ethics platform and the particularist perspective of narrative ethics. We will suggest an alternative (moderately particularist) approach to prioritization, which we dub a “principles plus medium-range narratives” approach. Notwithstanding the undeniably central role of principles in distributive justice, we claim that medium-range narratives concerning individuals or groups who stand to benefit or lose from ADHD prioritization practices should also be read or listened to and taken into account at all levels of priority-setting. These narratives are expected to ethically optimize clinical priority-setting, as well as that undertaken at local, regional, and national levels.

## Introduction

In Sweden, national, regional (county), and local (municipality) guidelines for open priority-setting in healthcare have been developed (Hofmann 2013; Carlsson 2010). However, priority-setting decisions may be predominantly and implicitly made at the micro (clinical) level (Ekerstad 2011). Obviously, national, regional, and local decision-making can act to limit the options available to individual clinicians. Mental healthcare users do not have many “opportunities to participate in decision/policy making about treatment” (Saxena 2007, 880), either individually or collectively. In matters of prioritization, participation is expected to be even rarer, due to institutionalized power imbalances between users and clinical, local, regional, or national decision-makers. In addition, healthcare-user participation should not be confused with public or citizen participation (Williamson 2014). However, as a surrogate for user participation, clinical, local, regional, or national decision-makers may be informed about user preferences by the findings of empirical (ethics) research.^1^ User preferences can thus, with a “detour,” be taken into consideration and given due weight in prioritization decision-making and policymaking.

Some narrative approaches to priority-setting in healthcare discuss physicians’ (e.g., Nordam 2005) and nurses’ (e.g., Langeland 2010) narratives. Yet, research on users’ narratives about priority-setting or other context-sensitive empirical ethical research (Musschenga 2005) on user preferences about matters of prioritization is lacking, particularly for ADHD. This lack of research on user preferences concerning prioritization in the context of ADHD is worrying since the cost-effective (Donnelly et al. 2004; Matthews, Nigg, and Fair 2003) but controversial psychopharmacological treatment of ADHD (Anijar and Gabbard 2005; Appelbaum 2005; Hoffmaster 2005; Kramer 2005; Krautkramer 2005; Litton 2005; Singh 2005a, 2005b; White 2005) has seen a significant rise over the last five to ten years (SMER 2015). It is even more a matter for concern, because the prevalence of ADHD is now estimated to be around 7 per cent (Thomas et al. 2015) and this figure has increased substantially within the past ten years, particularly in certain countries, such as the United States (Antshel et al. 2016). When we include parents or guardians, a substantial proportion of the total population has an interest in prioritization problems concerning individuals with ADHD. These problems may be articulated, as we will show in this paper, by “medium-range narratives,” concerning individuals or groups who stand to benefit or lose from ADHD prioritization practices. Roughly, these medium range narratives are epistemological constructs, deduced from one or more personal narratives, which bridge the gap between general rules and individual consequences. We will develop the concept of medium-range narrative in the remainder of this paper. Obviously, research on medium-range narratives is lacking. In this paper, we will start to bridge this research gap.

The following quote from a report by the Swedish Medical Ethics Council (SMER) may lift the veil on the ethical complexity of ADHD interventions somewhat:

It is not always obvious that drug treatment is the best method of treatment for a person with ADHD. There is much to suggest that psychosocial interventions in the form of psychotherapy and adjustments in the individual's environment can have a major effect on the individual’s ability to manage their ADHD-related problems. The great importance school environment for instance can have in terms of impact on individuals’ ADHD symptoms has been mentioned by several dialogue partners of SMER with insight into the subject. Adaptations in the school environment would thus likely reduce the need for drugs to treat children and adolescents. […] This can be expressed as an ethical conflict between individual needs and society’s ability to adapt and to allocate resources (SMER 2015, 63).^2^

In particular, SMER’s contention that adaptations in the school environment would be likely to reduce the need for drugs to treat children and adolescents reveals an ethical worry. The moral intuition we have here is that it is wrong to drug children and adolescents when other adaptations that could solve ADHD problems are feasible. Recent research reveals that “caregivers [of a child with ADHD] have different preferences for the intensity of medication use [and that] non-pharmacological aspects of care delivery, such as behavior management training and school accommodations, were preferred to evidence-based medications by caregivers” (Ng et al. 2017, 251). However, a thoroughgoing understanding of the consequences, user preferences, and moral responsibilities involved in what we may call the “ADHD-education-medication-prioritization-complex” is lacking. Personal narratives and what we propose to call medium-range narratives might contribute to this understanding.

In this paper, we aim to develop an ethical theoretical framework that can complement principle-based thinking with a narrative approach. Basically, narratives are “representations of events or series of events” (Abbott 2008, 13). Principle-based thinking or principlism is an influential way of mediating between different ethical convictions by concentrating on principles of medium range, presupposing that these principles are practically shared in all different ethical systems, no matter how they are founded (Beauchamp and Childress 2009). A narrative approach makes the participation of the personal perspective possible (apart from sheer lobbyism) and invites additional morally relevant information into the reflection process of prioritization. We present our suggestion in the particular case of having to choose between treatment interventions for ADHD and treatment interventions for other conditions or diseases, under circumstances of scarcity. In order to arrive at our model, we compare two distinct ethical approaches: a *generalist* (principles) approach and a *particularist* (narratives) approach. We conclude with a proposal for a *moderately particularist* (principles complemented with medium-range narratives) approach to prioritization, using the binding and at the same time perspective-broadening elements of medium-range narratives.

The structure of our paper is as follows. Firstly, we will give a summary of the “National Model for Transparent Prioritization in Swedish Health Care.” Our focus is on Sweden, because in this country prioritization in healthcare is uniquely governmentally regulated by the “ethics platform,” which is explained in the next section. Basically, it consists of three lexically ordered ethical principles: the human dignity principle, the needs-solidarity principle, and the cost-effectiveness principle. We hold the Swedish prioritization model in high regard, notwithstanding our suggestion for improvement. Secondly, we will give an overview of the main healthcare prioritization objects for ADHD (psychopharmacological and other treatment interventions). Thirdly, we will present a (fictional) scenario in order to test, successively, a principled and a narrative approach to prioritization. Fourthly, we will suggest an alternative approach to prioritization, which we will refer to as a “principles complemented with narratives” approach. This is an approach that enriches principled ethical decision-making in prioritization through ethical reflection on “personal” and “medium-range narratives.” Fifthly, we will end with concluding remarks.

## Prioritization in Swedish Healthcare: A Summary

The foundation of the Swedish national model for transparent prioritization (Broqvist et al. 2011) is called the ethics platform. The principles of the ethics platform are ordered in such a way that the cost-benefit principle is subordinate to both the human dignity principle and the needs-solidarity principle (Sandman 2015). “The principle of human dignity [should be taken into account] before the principle of needs-solidarity, and the same relation should apply between the principle of needs-solidarity and the principle of cost-effectiveness” (Broqvist 2018, 24). The parliamentary decision on the government bill addressing priorities in healthcare was taken in 1997 (Regeringens proposition 1996/97:60). Empirical evidence demonstrating a high level of national agreement with the three principles is largely absent. However, one study revealed that the three ethical principles were largely known by interviewed representatives from county councils and large professional groups as well as by interviewees from Swedish municipalities and were perceived as reasonable (Prioriteringscentrum 2007). Moreover, apart from discussions on details, there is almost no public debate questioning them.

These principles have been assigned by the Swedish government to be applied to priority-setting in healthcare in order for prioritization to be reasonable and just. In short, the parliamentary decision demands that “more resources will be allocated for the effective care of those in great need of care, provided there is a reasonable relationship between patient benefit and cost, and that human dignity is respected” (Prioriteringscentrum 2017, II). The aim of transparent prioritization is to “create acceptance among the population for the priorities and limitations that are unavoidable in health care, but also to give people the opportunity to react to and act upon the priorities set” (Broqvist et al. 2011, 11). The ethics platform was designed to include generally accepted principles because “the values underlying access to health services and the priorities set must be perceived to be fair by most of the population—in part to maintain the confidence and the will to publicly finance health care” (Broqvist et al. 2011, 12).

The *human dignity principle* is regarded as a principle of justice on the basis of formal equality; it “addresses factors that should not determine the priorities for care, e.g. personal characteristics and functions in society (e.g., talent, social position, income, age, gender)” (Broqvist et al. 2011, 13). The *needs-solidarity principle* means that “more of health care’s resources should be given to those in greatest need, those with the most severe conditions, and those with the lowest quality of life” (Broqvist et al. 2011, 13). It is a substantive interpretation of distributive justice and has clear practical consequences for prioritization between acute care, primary care, and life-threatening conditions. The *cost-effectiveness principle*, finally, means that “in choosing between different services or interventions, one needs to strive for a reasonable relationship between costs and effects, measured in terms of improved health and quality of life” (Broqvist et al. 2011, 14). Its neglect would result in a worse overall effect in a reality of limited resources; thus, it modifies the other prioritization principles in order to optimize the effect. However, the cost-effectiveness principle “should be applied at the group level, not at the individual level” (Ekerstad 2011, 12).

The principles of the ethics platform have been operationalized by the formulation of the so-called “components of the Swedish National Model for Transparent Prioritization.” These are: firstly, assessing the condition’s severity level; secondly, assessing the user benefits of the interventions; thirdly, assessing the interventions’ cost effectiveness; and, fourthly, transparently reporting the ranking itself, the reasoning behind the ranking and the consequences of the ranking. Moreover, an assessment of the quality of the knowledge base, concerning the benefits of the interventions and the cost effectiveness thereof, is demanded in the model, because, all other things being equal, interventions with stronger evidence should be prioritized over interventions with weaker evidence (Broqvist 2011).^3^

Although a condition of transparency is emphasized from the start of the Swedish National Model for Transparent Prioritization and can also be found in a recent report by the Swedish National Centre for Priorities in Health (Lund 2015), a condition of critical examination appears to be absent from this model. Practices of responsibility, including those that concern prioritization, should be found “under conditions of transparency *and*^4^ critical examination from many positions within those practices and outside of them” (Walker 2007, 20). The demand for critical examination in our context concerns the taking into account of medium-range narratives of mental healthcare users who are vulnerable to prioritization practices, for example, persons with ADHD. In theory, the demands of the principle of human dignity should be sufficient to safeguard equality. However, in practice prioritization practices may not be so disciplined. The ethics platform is an idealization that does not give any guarantee that unjust social realities, such as discrimination against marginalized groups within prioritization practices, can be counteracted or avoided. Nor does it offer sufficient tools to prevent such discrimination. “Idealizations that bypass [unacceptable] social realities” (Walker 2009, 5) can be criticized, held accountable and adjusted by using a naturalized bioethics approach.

The naturalism in ethics that we espouse […] is in the spirit of [a] self-reflexive, socially inquisitive, politically critical, and inclusive move toward an ethics that is empirically nourished but also acutely aware that ethical theory is the practice of particular people in particular times, places, cultures, and professional environments. (Walker 2009, 5)

In this paper, we suggest that medium-range narratives, as an instance of “naturalism in ethics,” present us with knowledge that may reveal deficiencies in principle-based prioritization. Moreover, they have the potential to be used as practical tools to adjust these deficiencies.

## Main Prioritization Objects for ADHD

Attention Deficit Hyperactivity Disorder (ADHD) is defined at a behavioural level, both by the WHO’s *International Classification of Diseases* (ICD 10) and the *Diagnostic Statistical Manual of Mental Disorders* (DSM-5) (American Psychiatric Association 2013). It “is […] defined by a persistent pattern of inattention and/or hyperactivity/impulsivity that interferes with functioning or development” (Instanes et al. 2016, 1). The American Psychiatrists Association distinguishes three subtypes of ADHD: inattentive, hyperactive/impulsive, and combined subtype (Rigler et al. 2016). Attention Deficit Hyperactivity Disorder is considered to be a highly heritable condition and a multitude of genes has been identified that have relevance for the condition (Matthews, Nigg, and Fair 2013). However, “both inherited and noninherited factors contribute to ADHD, and their effects are interdependent” (Thapar et al. 2013, 12). Both “psychiatric and non-psychiatric coexisting problems and clinical conditions have been described in ADHD patients. In particular, psychiatric comorbid conditions are recognized in both children and adults, and pose considerable clinical and public health challenges” (Instanes et al. 2016, 1). As the disorder is strongly defined by interaction with the social environment, its symptoms are not specific and can be caused by several different somatic and mental afflictions, and no reliable, significant, or validated tests for ADHD are available, the diagnosis is usually given in Sweden after a comprehensive (and resource-intensive) differential diagnosis including laboratory tests, neurological examination, comprehensive childhood history-taking (in the case of adults), and psychological and functional testing.

In terms of available therapy, a broad distinction can be made between psychopharmacological interventions and non-pharmacological (psycho-educative, psychological, environmental, and dietary) interventions. According to the European ADHD practice guidelines group, “methylphenidate, dexamphetamine and atomoxetine […] are effective drugs, and meta-analyses investigating their use have led to […] guidelines recommending their use as part of a comprehensive treatment program” (Graham et al. 2011, 17). However, the risks of these medications (Graham et al. 2011) are serious and need to be taken into account by clinicians. As the long-term use of central stimulants like the above-mentioned drugs, particularly for adults, has not yet been evaluated and as at least methylphenidate and amphetamine derivatives have addictive potential, special caution is required. Although the nature of these risks is not always known, “for many clinicians, the balance of risk against possible benefits of treatment will be seen as favorable in most cases” (Graham et al. 2011, 31). The guidelines group recognizes that, in the context of “what level of adverse effects is acceptable for a given treatment benefit for an individual person, [r]esponsive discussion with patients and their families is important” (Graham et al. 2011, 31). For adults with ADHD, pharmacological treatment with central stimulants like methylphenidate is the most common treatment (Bonvicini, Faraone, and Scassellati 2016). In Sweden, only psychiatrists are allowed to prescribe central stimulants for the indication of ADHD.

The first step in therapy, particularly for school children, is psycho-educative interventions, an adjusted environment with smaller groups, clear instructions and structures, electronic devices, and smart phone applications in order to remind patients of dates and duties. Sufficient and sufficiently frequent motion is also helpful. In addition, there are cognitive-behavioural therapies, relaxation training, and neurofeedback, weighted blankets for sleeping disorders, and restrictive elimination diets that are used in non-pharmacological therapies (Sonuga-Barke et al. 2013).

According to a recent review, “the majority of long-term outcomes of ADHD improve with all treatment modalities [pharmacological, non-pharmacological, and combination]. [However,] the combination of pharmacological and non-pharmacological treatment was most consistently associated with improved long-term outcomes and large effect sizes” (Arnold et al. 2016, 90).

In practice, the implementation of (state-of-the-art) multimodal treatment for ADHD appears to generate several prioritization problems, which appear on a daily basis in Sweden. Expensive prioritized medication, negotiations about possibly addictive medication, the need to encompass differential diagnosis for persons who have difficulties in getting or keeping a job, medical expert opinions for different authorities, and the exclusive responsibility for medication held by specialized psychiatry cause a lack of staff or limit the creation of organizational facilities for psycho-educative measures for other groups of psychiatric patients,^5^ illustrating the limiting impact of higher level (local, regional, or national) priority-setting decisions on the options available to individual psychiatrists.

Below, we present a fictional scenario that we use as a test case for different ethical approaches. The scenario is created in a possible future experiencing economic strain in order to emphasize the prioritization aspect of the principled approach taken by the Swedish ethics platform.

## Scenario

*Due to a deep economic recession, the government has decided that psychopharmacological interventions for ADHD have become too expensive. At the same time, alternative non-pharmacological interventions are equally deemed too expensive and/or are not regarded as being sufficiently evidence-based, so that other interventions for other conditions or diseases are prioritized in publicly funded healthcare. Emma, mother of a twelve-year-old boy Peter with ADHD, describes her own and her son’s experiences with school during the pre-recession and recession years: “We used to have good routines every day. It was not much of a problem getting Peter to school every day. He also liked going to school, at least he did not dislike it, the way he has done for a while now. He started complaining about the noise in the classroom, having to work in small groups with everybody talking all the time, so that he could not concentrate anymore. It was not such a big problem for him before because the classes were much smaller then, but after these so-called austerity measures taken by the government it has become much worse for Peter. First, they cut Peter’s behavioural therapy, but the situation became intolerable for both him and the school, at least for the teacher, when they decided not to compensate for the expense of Peter’s use of methylphenidate anymore. This meant that he had to do without the methylphenidate because we could not afford to pay for it ourselves. He immediately became much more restless in school, could hardly sit for any length of time, couldn’t concentrate on his work, and his results dropped dramatically because of that. He became angry because of the situation and started to show “acting out behaviour,” or so the teacher told us. They could not keep him in school any longer, and now he just sits in his room playing computer games the whole time. I don’t know if it’s because he’s going through puberty or because of the situation, or perhaps both, but he has also started to be disrespectful towards me and my husband. John, my husband, is away from home a lot of the time because of his work, so I had to scale down my full-time job to 50 per cent because of this new situation. I don’t want to leave Peter alone all day every day of the week. This means of course that I will not be able to get this promotion I aimed for, because there are other colleagues in our department who can outstrip me now. I don’t like it how things have turned out to be nowadays. I feel kept down most of the time, but I guess Peter must have that feeling as well, and perhaps even more so.”*

## A Principle-Based Approach to Prioritization

The approach of the ethics platform (Sandman 2015) can be thought of as a practical application of traditional principle-based bioethics. Although less relevant to the ethics platform, there are typical weaknesses in principle-based approaches. The scenario above nevertheless highlights problems with the current Swedish approach; that is, it fails to account for issues of moral responsibility.

Although principled approaches may be helpful and perhaps even necessary to “identify key values that clarify policy choices,” critics regard them as “insufficient because different principles […] lead to different conclusions and there is no consensus about which ones are correct” (Martin and Singer 2003, 60). The arbitrariness of resolving the problem of conflicting principles has been recognized as a severe criticism (Spielthenner 2017). In this respect, the ethics platform comes out more favourably than the influential approach of Beauchamp and Childress because of the stated hierarchy of the principles.

A second common criticism is that the principles “are too abstract to be directly used in actual decision making” (Martin and Singer 2003, 60). In the case of the ethics platform, this criticism loses much of its force, because the ethics platform is used very extensively, which contributes to its wider interpretation and also because of “established interpretations,” which can be found in reports from the Swedish National Centre for Priorities in Health.

A third criticism is that not everyone will necessarily agree with any given set of principles. For example, a strict egalitarian would disagree that cost-effectiveness should be a consideration, while a strict utilitarian may have reason to challenge the needs-solidarity principle.

A fourth criticism against bioethical principlism is that it is “thin on the facts [or] too little empirical knowledge is utilized in ethical deliberation and in formulating policy in areas of bioethical concern” (Parker 2009, 202). This complaint fails to some extent when applied to the ethics platform because, in using the national model, much attention is given to proven facts. Even the preferred methods used to value the known facts are highlighted. However, certain facts may be overlooked, as our scenario will point out.

Our scenario accentuates existing tensions between traditionally medical and more general societal factors, as well as the interconnections between the individual and the social sphere that are so obviously present in the case of ADHD. Given this scenario, the principle-based approach under increased economic and social pressure would have stopped paying, on all levels of priority-setting, both for central stimulants used to increase attention and self-control and for psycho-educative interventions and tools. Also, resources like extra rooms and extra personnel would have been withdrawn. This would not deny the *human dignity* of the pupils with ADHD; it would not be their social status, their gender, or similar traits that motivated the down-prioritization. Nevertheless, there would probably be more boys who would be without a decent education as a consequence of the retrenchment, because “in girls, internalising symptoms and inattentiveness are the more prominent ADHD symptoms, while boys tend to present with externalising symptoms such as impulsiveness and hyperactivity” (Kok et al. 2016). If the changed conditions led to a generally lower level of education for children with ADHD, their long-term social status could very well be affected as a side effect, even if not as a goal, of the changed school system.

In the principle-based approach to prioritization of the ethics platform, and based on the needs-solidarity principle, ADHD, as a chronic condition with mostly long-term and secondary effects in terms of suffering, will have to yield to more life-threatening conditions because these will be regarded as more urgent.

With regard to *cost effectiveness*, ADHD is a difficult case. It is mainly defined by social consequences, although its causes may be mainly biological. The disadvantageous consequences of the condition are, however, almost exclusively social; only a very severe degree of ADHD causes primary suffering. Many therapeutic approaches address the social environment, both in training the individual to adjust to society (pharmaceutically and through education) and in adjusting society to the needs of the person with ADHD. It is methodologically extremely difficult to compare the different therapeutic approaches because so many of the factors are not easily created in experimental forms. For example, having classes of sixty instead of thirty pupils is probably a disadvantage for all pupils, and there are not many parents who would allow their children to suffer obvious disadvantages that could affect their whole social life just in order to generate reliable knowledge about the consequences in experimental circumstances. Furthermore, it is impossible to test those environmental factors in a double-blind way. With regard to evidence-based, reliable knowledge, the more a condition is complex and socially caused or defined, the higher the risk that the cost-effectiveness is difficult to calculate. Pharmaceutical therapies have the easiest access to evidence-based medicine standards, and at the same time they are most easily distributed in a just way. This may be a background reason as to why pharmacological treatment with central stimulants like methylphenidate has a tendency to be favoured in comparison to other interventions, even if it is not at all clear whether they are the most cost-effective, particularly if not combined with social and psychological efforts. If, as a result of considerations of the human dignity principle, only one single intervention for children were after all to be allowed in our scenario, it would probably be the prescription of methylphenidate.

One type of consequence that is difficult to address in the ethics platform is the following. The dynamic of the scenario would probably change the intra- and interpersonal balance of parental responsibility. If society no longer supports the treatment of ADHD and privatizes the disadvantages of limited resources for education, the equality of human dignity (which is the highest prioritization factor) is challenged. It would be left exclusively to the parents’ responsibility to help their child with ADHD get support and education even under these circumstances. Those who can afford the high price of central stimulants could perhaps give their children the same opportunities in school as other children. They could pay privately for psycho-educative therapy and auxiliary devices. Ultimately, they could even pay for private education. Those who cannot afford all this would have to deal with a child who is excluded from school and either stays at home alone or with one of the parents. In a society experiencing gender inequality, even a relatively equal one,^6^ it will too often be the case that the mother is expected to shift her focus of responsibility. In such a case, parental responsibility is adversely affected by resource distribution processes and could exacerbate existing imbalances between the genders.

This scenario shows that responsibility for the distribution of resources within publicly funded professional care is morally interconnected with the distribution of responsibilities in intimate, domestic, or familial—“private”— contexts. This moral interconnectedness presumably exists not only in situations of crisis but also under “normal” circumstances. It follows that the consequences of the distribution of resources for the distribution of responsibilities need to be known in order to arrive at a high-quality ethical decision and/or policymaking in prioritization. In general, justice-based accounts tend to obscure these “private” forms of responsibility (Martin and Singer 2003) and therefore this morally relevant information is often ignored.

A relevant criticism against a principle-based approach in ethics, in our context, is that:

It ignores the often unchosen, discretionary responsibilities of those who care for particular others, often dependent and vulnerable, in intimate, domestic, or familial—“private”—contexts. It slights relations of interdependence centered on bonds of affection and loyalty whose specific histories set varying terms of obligation and responsibility. It obscures the particularity of moral actors and relations by emphasizing universality, sameness, and repeatability, excluding or regimenting emotional experience. (Walker 2007, 58)

One might question this criticism by stating that the ethics platform is intended to address the distribution of resources within publicly funded professional care, which makes the position of these forms of responsibility less clear. However, the distribution of resources within publicly funded care can adversely affect the responsibilities of those who care for particular others in a direct manner, which was one of the moral saliences in our scenario. One might even broaden the issue by asking to what degree healthcare should be used for social engineering (to correct gender imbalances), especially since there is a risk that this will be bought at the cost of healthcare for other patients. However, such a wider issue, although important, does not detract from our argument that the distribution of healthcare resources by clinical, local, regional, or national priority-setting decision-makers may aggravate gender imbalances in the first place and consequently boost the need for social engineering to correct these gender imbalances. How to pay for this social engineering is beyond the scope of our argument in this paper.

In the next step, we will investigate how a narrative (particularistic) account can deal with the prioritization challenge.

## A Narrative Approach to Prioritization

American philosopher Margaret Urban Walker understands narratives to be “stories that show how a situation comes to be the particular problem it is, and that explore imaginatively the continuations that might resolve the problem and what they mean for the parties involved” (Walker 2007, 72). Narratives are “the basic form of representation for moral problems. We need to know who the parties are, how they understand themselves and each other, what terms of relationship obtain, and perhaps what social or institutional frames shape their options” (Walker 2007, 75).

In Walker’s ethics of responsibility, narratives of relationship, narratives of moral identity, and narratives of value are central to the representation of moral problems:

A *narrative of relationship* is a story of the relationship’s acquired content and developed expectations, its basis and type of trust, and its possibilities for continuation. […] The agent’s own *narrative of moral identity* is a persistent history of valuation that can be seen in a good deal of what a person cares for, responds to, and takes care of. […] The narratives of relationship and identity inevitably intertwine. […] Yet there is a third kind of narrative that spans and supports both of these. It is a history of our shared understandings of what kinds of things, relationships, and commitments really are important, and what their relative importance is. This is the *narrative of moral values*. (Walker 2007, 118–119)

According to Walker, the basic claim about the structure of our responsibilities is this:

Specific moral claims on us arise from our contact or relationship with others whose interests are vulnerable to our actions and choices. […] We are *obligated to respond* to particular others when circumstance or ongoing relationship render them especially, conspicuously, or peculiarly dependent on us. (Walker 2007, 113)

If we think of our scenario of reduced public extra resources for children with ADHD, we would have to find particular solutions to each particular life history. Peter’s family has responded to the situation according to their own opportunities: as long as possible, they tried to do without medicine or psycho-educative therapy and hoped that Peter would handle the school situation anyway; they tried to pay for the therapy on their own but could not afford it, then Peter was excluded from school and at first he stayed at home on his own, which they judged to be an intolerable situation. So finally, Emma bore the costs of handling the situation and stayed at home against her own wishes and career plans. If we regard this scenario as an individual narrative from the first-person perspective, it elucidates the problem and the ethical demands, but certain social or general ethical demands do not follow. Each fate, for children and their families in a similar situation, has to be resolved in a particular way. In certain cases, there may be opportunities to work with engaged teachers and solidary classmates and communal engagements to help those children with special needs in spite of the constrained general circumstances. Certain families might have a good private network of friends and grandparents who can help in a good way even though the state has put children with ADHD at a disadvantage. Other families may have economic resources themselves in order to pay for the individual child’s needs. As care exists in particular relationships, there will be particular solutions to the problem, some of them better, some of them worse than extended public support.

An obvious criticism of (partial) narrative thinking is that the resulting solutions to the problem will be multiple, unequal, and unjust. Traditionally, narrative medical ethics is used to complement an existing healthcare system that is focused on facts and evidence-based guidelines, on the general factors of disease, and on the statistically most promising therapy (Brody and Clark 2014; Frank 2014). Indeed, all kinds of therapy have to be adjusted to particular patients in particular circumstances, and this needs as much attention and education as learning the general knowledge base. Although it is problematic to base national decisions on individual narratives because of volume and high selectivity, which is a major limitation of the narrative approach, individual narratives remain excellent tools to help broaden one’s perspective to others’ subjective experiences and perspectives (Ahlzén 2010). Are individual narratives as useful to political decision makers, particularly in prioritization, as for “doctors in the making” (Poirier 2009)? After all, they also have to find a way to combine the general with the particular, and at least the second criterion of the ethics platform, comparing the needs of patients, is hardly understandable without reference to particular suffering. But the cost-effectiveness can also be understood with regard to particular, not only to average, effects. Even if a therapy (A) for cancer is on average cheaper than and as effective as a more expensive one (B), it may be worthless in an individual case and thus much too expensive in terms of both money and side effects. The more expensive alternative (B)—if effective in this case—may be more cost-effective with regard to the particular case. Should wise policymakers therefore use individual narratives instead of general statistics as the basis for their decisions?

Another major limitation of the narrative approach is that it provides a voice only to those patients who are the subjects of prioritization decisions. In our scenario, although Emma’s voice is heard (and, by imperfect proxy, Peter’s), those patients who will suffer a loss if Peter’s treatment is funded (e.g. because their own treatment cannot be funded as a result) are not. In the context of prioritization, clinical, local, regional, or national decision-makers can become so concerned with individual patients that they find it difficult to see the whole system, in particular, the needs of other patients. This can be seen when rules are created after scandals that draw huge media attention, with emotionally moving pictures, for example, of individual cute children or pets. Often, the new decisions regulate situations as presented in the public but without a rational balancing of all the relevant factors and all the possible parties involved in the consequences of the new procedures. With regard to narratives of particular patients who are evaluated against each other, it is by no means clear that those in greatest need are the best or most convincing narrators. Those who are suffering the most might be silenced by their own needs, unable, for example, to leave their rooms to be in time for a hearing or to use the technical equipment required to reach an internet forum. As a result, the society, even if individually caring, might become more unjust and guided by the opportunities for personal relationships (or media attention) alone.

On the other hand, an obvious criticism of (impartial) justice-based thinking is that clinical, local, regional, or national decision-makers can become so concerned with the whole system that they find it difficult to see the individual patients. As a result, they may take inhumane, though equal, decisions. In the case of ADHD, which is a diagnosis that is in many ways socially defined and shaped, there is a risk that social factors which traditionally are not in the domain of healthcare, healthcare budget, and the prioritization platform will be overlooked.

We will now attempt to describe a promising position that helps to bridge between the particular and the general perspectives.

## A Principles Plus Medium-Range Narratives Approach to Prioritization

The two previous sections have revealed the problems faced by the principle-based approach in attempting to cover all the complexities and particularities of the consequences of a rationing decision. A narrative prioritization approach, on the other hand, runs the risk that the most eloquent persons with the most effective networks will get the best opportunities, at the cost of both the average “normal” people and those who are disadvantaged in terms of networks and advocates. Is it possible to combine the advantages of both approaches without doubling the disadvantages? This would mean a moderate particularism, which allows morally relevant features to be identified by principles or other moral rules “but denies that moral rules can identify every moral feature” (van Willigenburg 1999, 58). The remaining moral features may be discovered by listening to, reflecting upon, and taking into account “medium-range narratives” in the process of moral decision-making.

There exists, however, an apparently insoluble tension (in theory) between partial and impartial approaches to ethics, that can be bridged (in practice) by allowing moderate partiality in prioritization contexts. From an ethics of care point of view, there are at least “three different ways how partiality can legitimately be considered in a basically general context” (Nortvedt, Hem, and Skirbekk 2011, 192). In terms of our scenario, which confronts us with the result of a prioritization based on a principled decision, we miss an opening for individual or social adjustments that involves these three ways: (a) a consideration of extreme circumstances, (b) the acceptance of the inevitable connectedness of caregiver/teacher’s and patient/pupil’s integrity, and (c) space for individual and personal ethical caregiving.

We assume that a principle-based background is inevitable for just prioritization at all levels, that is, clinical, local, regional, and national. However, if we give space to individual narratives and arguments, extreme consequences in single cases can be revealed and mended (a). For example, there might be the possibility of paying for central stimulants in certain cases (extremely severe ADHD or extremely poor parents, at the cost of patients with other diseases).

If we consider that the integrity of teacher and pupil is essentially connected (b) and, in our scenario, broken because Peter is unable to adjust to the new setting in school, which probably implies a feeling of total failure on both sides, as a pupil and as a teacher who loses her pupil, we might allow overall cost-neutral solutions that make it easier for the teachers to keep Peter in school. The general class size could be reduced according to the number of pupils with special needs (at the cost of average kids), or special classes could be offered that are adjusted to the special needs, even if they perhaps cannot give the same kind of education as average children get. Both decisions would cause a deterioration in the situation of other children, but this had to be balanced individually against the advantage gained by Peter and his teachers in preserving their respective integrity as teacher and pupil.

Finally, it would be important to explicitly allow for individual solutions that adjust the general rules and intentions to the particular situation, in discussion with teachers, parents, classmates, and the child in question (c).

Independently of an ethics of care point of view, the basic concept of equity might also be a useful framework for legitimating partiality. In this case one starts with “the principle that like cases should be treated as like and unlike as unlike […]. That is, patients who are alike in relevant respects should be treated the same, and those who are unlike in relevant respects should be treated in appropriately different ways” (Clark and Weale 2012, 306).

Finding individual solutions is resource-consuming in itself, but it is inevitable when applying general rules in practice, even if it may be easier in average cases. Aristotle called this capacity practical wisdom (phronesis), Immanuel Kant called it “Urteilskraft,” but no general rule can be wisely or justly applied without it. It is important to encourage this attitude to rules and laws, because the tendency to follow the words but not the intention of rules—legal positivism instead of ethically enlightened legalism—is devastating to society. With regard to Peter in our scenario, as well as many other children with similar or other problems resulting from rationing, many different individual solutions should be discussed and analysed, not only economically but also with regard to their ethical and social costs, and a certain variability in all principle-based equality should be maintained.

Still, it remains unclear how a wise legislator at a national level can foresee certain effects of prioritization decisions that are not easily described by either general rules of justice or by individual case stories (Charon 2006). Our scenario happens to highlight examples of social, not only individual, costs: one of the parents is forced to stay at home—and it is probably the parent who is earning less money and/or is more inclined to sacrifice personal career options for the sake of the family. Traditional role models may contribute to these factors. If only presented as an individual narrative, this should be reason to consider increased support in this individual case but the social dimension would be missed. Actually, our scenario *is not* just an individual, empirical narrative. It encompasses a fictive, individual narrative, but this is only a part of it. The story is simultaneously a thought experiment, an imaginative expansion of actual problems and tensions into a near future with the intention of pointing out certain dangers and implied directions of medical/school political rationing decisions. It is what we would like to call a “medium-range narrative.”

## Medium-Range Narratives as a Tool between the General and the Particular

The term “medium-range narrative” is inspired by Beauchamp and Childress who invented the “medium-range principles” approach in order to mediate between all kinds of different philosophical and religious ethical systems (Beauchamp and Childress 2009). Our intention is to find and use a tool to bridge the gap between general rules and decisions in the public sphere and the consequences of those decisions at a personal and individual level. This reduces neither the necessity for principled bases for decision-making nor the importance of individual experiences and their representation in personal narratives which underlie critical examination of the decisions and their consequences. As a matter of fact, there are several methods for applying personal experiences in order not to neglect important aspects of the decisions. Having a balanced decision-making board with regard to gender, age, and groups that are concerned in the decision is one of these methods. In addition to this, and perhaps partly in place of it, it could be useful to apply imaginative expansions of the limited experiences of the decision-makers.

In ethical theory, different kinds of narratives are commonly used, with the narrative taking a more or less important role. In casuistry, for example, ethical problem cases are compared to clear-cut cases (*paradigms*) using general principles of reasoning. Within Hans Jonas’s *The Imperative of Responsibility*, *worst case scenarios* are used in order to enlarge the sphere of personal responsibility and respective caution (Jonas 1984). In analytic philosophy, *thought experiments* are popular in order to concentrate on certain crucial aspects of situations that are otherwise too complex. In public ethical debates*, tragic stories* are commonly used, both in pictures and words, in order to engage moral emotions. In narrative ethics, *first-person singular narratives* are used with the aim of re-introducing the individual subject into the objectivist world of science and generalist philosophy.

If we advocate the use of medium-range narratives in ethical (political) decision-making, some of these kinds of narratives could be included. Our point in emphasizing the medium range is the bridge between the individual and the general sphere. Thus, medium-range narratives are stories (a) about the individual (b) within relations (c) within society. They take their place between other similar stories, between other different stories, between personal experience and rules and laws, and within cultures and subcultures. Medium-range narratives can be realistic (e.g. taken from qualitative research), fictional, or hypothetical (like our scenario).

We used our scenario in order to clarify certain aspects of the Swedish ethics platform that might be missed from the purely principled perspective of justice. We chose the example of ADHD as deeply rooted in social contexts, and we chose a context of growing scarcity in order to focus on the prioritization context of the ethics platform, pertaining to the clinical, local, regional, and national level of priority-setting, and also in order not to get stuck in the empirical detail of questions where reliable evaluations are still missing. With the help of our scenario, we were able to analyse the strengths and weaknesses of the generalist principle-based perspective of the ethics platform and the particularistic perspective of narrative ethics. Both approaches showed themselves to be useful but not sufficient in order to meet the complexities of the addressed situation. The spheres that can be easily missed are broader social aspects that the individual story mentions but does not recognize as a social and culturally shaped problem.

In short, the potential of medium-range narratives is that they can:

- integrate different perspectives
- fill a gap between the personal and the public
- allow experimenting with different scenarios and the possible consequences of choices and decisions.

As such, medium-range narratives do not represent a new ethical theory, nor are they apt as an isolated method of decision-making. They are, however, a useful intellectual tool in ethical (political) decision-making that should not be left only to artists or playful bystanders.

## Concluding Remarks

In this paper, we have critically argued that the generalist approach underlying the ethics platform, a guide for priority setting in Swedish healthcare at clinical, local, regional, and national levels of priority setting is insufficient as a framework for empirical ethical research on the consequences of prioritization, user preferences, and moral responsibilities, at least in the context of ADHD. We have argued for the superiority (again, at least in the context of ADHD) of a moderately particularistic approach to prioritization. More concretely, we have introduced a “principles plus medium-range narratives” approach to prioritization. Reflecting on our imaginary scenario, we saw that, on the one hand, an exclusively principle-based approach tends to ignore problems of (re-)distributing moral responsibilities. An exclusively narrative approach, on the other hand, charting moral responsibilities through narratives of moral identity, relationships, and moral values, would correct this defect of a principle-based approach but runs the risk of misinterpreting the centrality of principles in distributive justice (in Sweden the principles of the ethics platform), which should take priority in the domain of prioritization of healthcare interventions. Our suggestion of medium-range narratives as a complementary tool to principle-based prioritization is expected in particular to ethically optimize prioritization decision-making and policymaking at all levels of priority-setting.

## Footnotes

1 Preferences are best identified empirically; both quantitative and qualitative research is necessary and relevant. Such research can be ethical either by respecting the perspectives of relevant parties who have no opportunity to be directly involved in the ethical decision-making process (see Habermas 1991 on discourse ethics) or by aiming at the ethical aspect of prioritization, asking for arguments as to why some measures ought to be prioritized over others.

2 Translation: PJ.

3 This clarification might seem a harmless reformulation of cost-effectiveness, but it implies a general prioritization of less complex interventions (e.g. easier and cheaper to test), as the gold standard of evidence-based medicine is designed according to pharmacological research, not to the requirements of clinical research or the evaluation of psychosocial interventions. It has recently been seriously questioned, as its results have been shown to be significantly influenced by the interests of the sponsor, despite all methodological efforts to provide pure, disinterested evidence (Ahn et al. 2017; Johansson 2017).

4 Our emphasis.

5 Personal experiences of PG in a regional general psychiatric unit with both out-patient and in-patient hospital ward in Östergötland, Sweden, which is shared independently by colleagues from many other parts of the country.

6 See http://hdr.undp.org/en/composite/GII. Accessed November 6, 2017. Sweden’s gender inequality index for 2015 is 0.048. Sweden is ranked #4, only surpassed by the Netherlands (0.044), Denmark (0.041), and Switzerland (0.040).

## References

[CR1] Abbott HP (2008). *The Cambridge introduction to narrative*.

[CR2] Ahlzén R (2010). Why should physicians read? Understanding clinical judgment and its relation to literary experience.

[CR3] Ahn R, Woodbridge A, Abraham A (2017). Financial ties of principal investigators and randomized controlled trial outcomes: Cross sectional study. BMJ.

[CR4] American Psychiatric Association (2013). *Diagnostic and statistical manual of mental disorders, fifth edition (DSM-5)*.

[CR5] Anijar K, Gabbard D (2005). Authentic faux diamonds and attention deficit disorder. The American Journal of Bioethics.

[CR6] Antshel KM, Zhang-James Y, Wagner KE, Ledesma A, Faraone SV (2016). An update on the comorbidity of ADHD and ASD: A focus on clinical management. Expert Review of Neurotherapeutics.

[CR7] Appelbaum PS (2005). Psychopharmacology and the power of narrative. The American Journal of Bioethics.

[CR8] Arnold LE, Hodgkins P, Caci H, Kahle J, Young S (2016). Effect of treatment modality on long-term outcomes in attention-deficit/hyperactivity disorder: A systematic review. Focus.

[CR9] Beauchamp TL, Childress JF (2009). *Principles of biomedical ethics*.

[CR10] Bonvicini C, Faraone SV, Scassellati C (2016). Attention-deficit hyperactivity disorder in adults: A systematic review and meta-analysis of genetic, pharmacogenetic and biochemical studies. Molecular Psychiatry.

[CR11] Brody H, Clark M (2014). Narrative ethics: A narrative. Hastings Center Report.

[CR12] Broqvist M (2018). *Asking the public: Citizens’ views on priority setting and resource allocation in democratically governed healthcare*.

[CR13] Broqvist, M., M. Branting Elgstrand, P. Carlsson, K. Eklund, and A. Jakobsson, A. 2011. *National model for transparent prioritisation in Swedish health Care* (Revised Version). Linköping: Swedish National Centre for Priorities in Health. Linköping: National Center for Priority Setting in Health Care.

[CR14] Carlsson P (2010). Priority setting in health care: Swedish efforts and experiences. Scandinavian Journal of Public Health.

[CR15] Charon R (2006). *Narrative medicine: Honoring the stories of illness*.

[CR16] Clark S, Weale A (2012). Social values in health priority-setting: A conceptual framework. Journal of Health Organization and Management.

[CR17] Donnelly M, Haby MM, Carter R, Andrews G, Vos T (2004). Cost-effectiveness of dexamphetamine and methylphenidate for the treatment of childhood attention deficit hyperactivity disorder. Australian and New Zealand Journal of Psychiatry.

[CR18] Ekerstad N (2011). *Micro level priority setting for elderly patients with acute cardiovascular disease and complex needs*.

[CR19] Frank AW (2014). Narrative ethics as dialogical story-telling. Hastings Center Report.

[CR20] Graham J, Banaschewski T, Buitelaar J (2011). European guidelines on managing adverse effects of medication for ADHD. European Child & Adolescent Psychiatry.

[CR21] Habermas J (1991). *Moral consciousness and communicative action*.

[CR22] Hoffmaster B (2005). “Real” ethics for “real” boys: Context and narrative in bioethics. The American Journal of Bioethics.

[CR23] Hofmann B (2013). Priority setting in health care: Trends and models from Scandinavian experiences. Medicine Health Care and Philosophy.

[CR24] Instanes JT, Klungsøyr K, Halmøy A, Fasmer OB, Haavik J (2016). Adult ADHD and comorbid somatic disease: A systematic literature review. Journal of Attention Disorders.

[CR25] Johansson S (2017). När evidensens grundval sätts i skakning [When the foundation of evidence is shaken]. Läkartidningen.

[CR26] Jonas H (1984). *The imperative of responsibility. In search of an ethics for the technological age*.

[CR27] Kok FM, Groen Y, Fuermaier ABM, Tucha O (2016). Problematic peer functioning in girls with ADHD: A systematic literature review. PLoS ONE.

[CR28] Kramer PD (2005). Real impairments, real treatments. The American Journal of Bioethics.

[CR29] Krautkramer CJ (2005). Beyond creativity: ADHD drug therapy as a moral damper on a child's future success. The American Journal of Bioethics.

[CR30] Langeland K, Sørlie V (2010). Ethical challenges in nursing emergency practice. Journal of Clinical Nursing.

[CR31] Litton P (2005). ADHD, values, and the self. The American Journal of Bioethics.

[CR32] Lund K (2015). *Socialstyrelsens nationella riktlinjer och kommunens prioriteringar [National Board of Health’s national guidelines and the municipality's priorities].* Reportapport 2015:4.

[CR33] Martin D, Singer P (2003). A strategy to improve priority setting in health care institutions. Health Care Analysis.

[CR34] Matthews, M., J.T. Nigg, and D.A. Fair. 2013. Attention deficit hyperactivity disorder. In *Current Topics in Behavioral Neuroscience* 16: 235−266. 10.1007/7854_2013_249 PMC4079001 24214656

[CR35] Musschenga AW (2005). Empirical ethics, context-sensitivity, and contextualism. The Journal of Medicine and Philosophy.

[CR36] Ng X, Bridges JFP, Ross MM (2017). A latent class analysis to identify variation in caregivers’ preferences for their child’s attention-deficit/hyperactivity disorder treatment: Do stated preferences match current treatment?. Patient.

[CR37] Nordam A, Sørlie V (2005). Prioritisation and quality of care for elderly people—Male physicians’ narratives. Vård i Norden.

[CR38] Nortvedt P, Hem MH, Skirbekk H (2011). The ethics of care: Role obligations and moderate partiality in health care. Nursing Ethics.

[CR39] Parker M (2009). Two concepts of empirical ethics. Bioethics.

[CR40] Poirier S (2009). *Doctors in the making. Memoirs and medical education*. Iowa: University of Iowa Press.

[CR41] Prioriteringscentrum. 2007. *Vårdens alltför svåra val? Kartläggning av prioriteringsarbete och analys av riksdagens principer och riktlinjer för prioriteringar i hälso- och sjukvården [Healthcare’s too difficult choice? Mapping priority work and analysis of parliament’s principles and guidelines for priorities in healthcare].* Prioriteringscentrum Rapport March 2007.

[CR42] Prioriteringscentrum. 2017. *Nationell modell för öppna prioriteringar inom hälso- och sjukvård – ett verktyg för rangordning [National model for open priority setting within healthcare—A tool for ranking]*. Prioriteringscentrum Rapport 2017: 2.

[CR43] Regeringens proposition 1996/97:60. Prioriteringar inom hälso- och sjukvården [Priorities in healthcare]. http://www.riksdagen.se/sv/dokument-lagar/dokument/proposition/prioriteringar-inom-halso%2D%2Doch-sjukvarden_GK0360/html. Accessed November 15, 2018.

[CR44] Rigler T, Manor I, Kalansky A, Shorer Z, Noyman I, Sadaka Y (2016). New DSM-5 criteria for ADHD—Does it matter?. Comprehensive Psychiatry.

[CR45] Sandman L (2015). *Vilken vägledning ger den etiska plattformen för prioriteringar i konkreta prioriteringssituationer?[What guidance does the ethical platform offer for setting priorities in specific situations? A review of problems in interpretation and implementation]*. Rapport 2015:3.

[CR46] Saxena S (2007). Resources for mental health: Scarcity, inequity, and inefficiency. The Lancet.

[CR47] Singh I (2005). Will the “real boy” please behave: Dosing dilemmas for parents of boys with ADHD. The American Journal of Bioethics.

[CR48] Singh, 2005b. Response to commentators on “will the ‘real boy’ please behave: Dosing dilemmas for parents of boys with ADHD”. *The American Journal of Bioethics* 5(3): W10−W12. 10.1080/15265160500194410 28570166

[CR49] SMER (Statens medicinsk-etiska råd) (2015). *ADHD—etiska utmaningar [ADHD—Ethical challenges]*. Smer rapport 2015:2.

[CR50] Sonuga-Barke EJS, Brandeis D, Cortese S (2013). Nonpharmacological interventions for ADHD: Systematic review and meta-analyses of randomized controlled trials of dietary and psychological treatments. The American Journal of Psychiatry.

[CR51] Spielthenner G (2017). The principle-based method of practical ethics. Health Care Analysis.

[CR52] Thapar A, Cooper M, Eyre O, Langley K (2013). Practitioner review: What have we learnt about the causes of ADHD?. The Journal of Child Psychology and Psychiatry.

[CR53] Thomas R, Sanders S, Doust J, Beller E, Glasziou P (2015). Prevalence of attention-deficit/hyperactivity disorder: A systematic review and meta-analysis. Pediatrics.

[CR54] van Willigenburg, T. 1999. Guidance by moral rules, guidance by moral precedents. In *Reasoning in ethics and law: the role of theory, principles and facts*, edited by A.W. Musschenga and W.J. van der Steen, 49−62. Aldershot: Ashgate.

[CR55] Walker MU (2007). *Moral understandings: A feminist study in ethics*.

[CR56] Walker, 2009. Introduction: Groningen naturalism in bioethics. In *Naturalized bioethics: Toward responsible knowing and practice,* edited by H. Lindemann, M. Verkerk, and M.U. Walker, 23−41. Cambridge: Cambridge University Press.

[CR57] White GB (2005). Splitting the self: The not-so-subtle consequences of medicating boys for ADHD. The American Journal of Bioethics.

[CR58] Williamson L (2014). Patient and citizen participation in health: The need for improved ethical support. The American Journal of Bioethics.

